# Unraveling Membrane-Disruptive Properties of Sodium Lauroyl Lactylate and Its Hydrolytic Products: A QCM-D and EIS Study

**DOI:** 10.3390/ijms24119283

**Published:** 2023-05-25

**Authors:** Negin Gooran, Sue Woon Tan, Bo Kyeong Yoon, Joshua A. Jackman

**Affiliations:** 1 School of Chemical Engineering and Translational Nanobioscience Research Center, Sungkyunkwan University, Suwon 16419, Republic of Korea; 2 School of Healthcare and Biomedical Engineering, Chonnam National University, Yeosu 59626, Republic of Korea

**Keywords:** lactylate, phospholipid membrane, supported lipid bilayer, quartz crystal microbalance–dissipation, electrochemical impedance spectroscopy

## Abstract

Membrane-disrupting lactylates are an important class of surfactant molecules that are esterified adducts of fatty acid and lactic acid and possess industrially attractive properties, such as high antimicrobial potency and hydrophilicity. Compared with antimicrobial lipids such as free fatty acids and monoglycerides, the membrane-disruptive properties of lactylates have been scarcely investigated from a biophysical perspective, and addressing this gap is important to build a molecular-level understanding of how lactylates work. Herein, using the quartz crystal microbalance–dissipation (QCM-D) and electrochemical impedance spectroscopy (EIS) techniques, we investigated the real-time, membrane-disruptive interactions between sodium lauroyl lactylate (SLL)—a promising lactylate with a 12-carbon-long, saturated hydrocarbon chain—and supported lipid bilayer (SLB) and tethered bilayer lipid membrane (tBLM) platforms. For comparison, hydrolytic products of SLL that may be generated in biological environments, i.e., lauric acid (LA) and lactic acid (LacA), were also tested individually and as a mixture, along with a structurally related surfactant (sodium dodecyl sulfate, SDS). While SLL, LA, and SDS all had equivalent chain properties and critical micelle concentration (CMC) values, our findings reveal that SLL exhibits distinct membrane-disruptive properties that lie in between the rapid, complete solubilizing activity of SDS and the more modest disruptive properties of LA. Interestingly, the hydrolytic products of SLL, i.e., the LA + LacA mixture, induced a greater degree of transient, reversible membrane morphological changes but ultimately less permanent membrane disruption than SLL. These molecular-level insights support that careful tuning of antimicrobial lipid headgroup properties can modulate the spectrum of membrane-disruptive interactions, offering a pathway to design surfactants with tailored biodegradation profiles and reinforcing that SLL has attractive biophysical merits as a membrane-disrupting antimicrobial drug candidate.

## 1. Introduction

Antibiotic-resistant bacteria are a major global issue for healthcare and sustainability, and ongoing regulatory actions such as limiting antibiotic use in food and feedstuffs are prompting the development of antibiotic alternatives [1,2]. While antibiotics often inhibit specific bacterial enzymes, there is growing interest in the use of more broadly acting antimicrobial peptides and lipids that can disrupt bacterial cell membranes [3], in turn abrogating cell viability and causing cell death in some cases [4,5]. Among different candidates, antimicrobial lipids such as medium-chain, saturated fatty acids and monoglycerides have received extensive attention due to their membrane-disruptive activities that work against not only various types of bacteria but also membrane-enveloped viruses and other microorganisms [6,7,8]. Structure–function relationship studies have mainly looked at how antimicrobial lipid properties such as chain length and headgroup charge affect antimicrobial activities [9], while there is growing interest to characterize the biophysical properties of antimicrobial lipids, i.e., their membrane-disruptive effects, using surface-sensitive measurement approaches that provide insight into compound potency and corresponding interaction processes. These efforts have shed mechanistic light on the biophysical properties of medium-chain fatty acids and monoglycerides, while highlighting the opportunity to extend such approaches to characterize additional classes of biologically significant molecules, such as amphipathic quorum-sensing signaling molecules [10], as well as industrially significant surfactants and detergents that bear resemblance to fatty acids and monoglycerides [11]. 

As esterified adducts of fatty acids and lactic acid (LacA), lactylates are widely used as food additives and have recently received heightened attention for industrial applications such as animal agriculture due to membrane-disruptive antimicrobial properties [12,13]. In particular, it has been reported that lactylates containing 12- and 14-carbon-long saturated fatty acid chains are able to potently inhibit *E.coli* bacteria in piglets [14] and have also demonstrated in vitro antibacterial activity against *C. perfringens* bacteria at levels that are >100 times more potent than free fatty acids alone [15]. Of particular note, sodium lauroyl lactylate (SLL) is a lactylate derivative of 12-carbon-long, saturated lauric acid (LA) and has been reported to be more effective at inhibiting *C. perfringens* infection in poultry compared with free fatty acids and mono- and diglycerides [15]. In terms of industrial applicability, lactylates such as SLL also have a higher degree of hydrophilicity and hence greater solubility in water than corresponding fatty acids [16,17]. On the other hand, while the effect of free SLL on human cell membranes has not been reported, SLL-based liposomal formulations for drug delivery applications have been shown to modestly reduce human keratinocyte cell proliferation in a dose-dependent manner [18], suggesting that SLL might cause some degree of human cell membrane disruption as well and motivating a deeper understanding of how SLL disrupts phospholipid membranes from a molecular-level biophysical perspective. Addressing this gap is particularly important because lactylates such as SLL are susceptible to hydrolytic cleavage, e.g., by enzymes, in physiological environments that would likely impact membrane-disruptive properties [19] (for comparison, see other examples of related molecules that are less susceptible to hydrolytic cleavage [20]).

**Figure 1:**
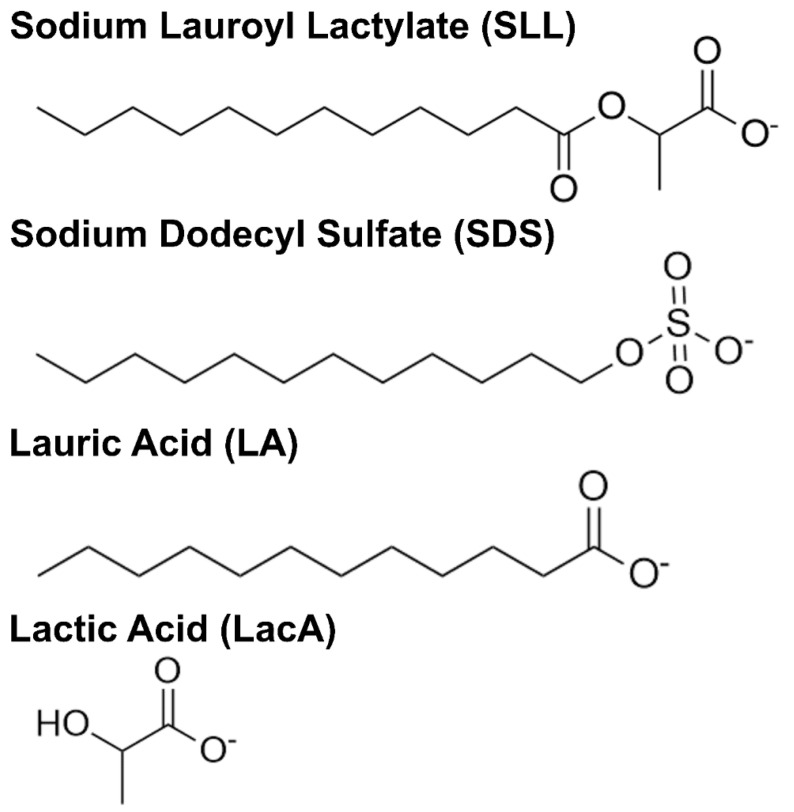
Molecular structures of compounds used in this study. Where applicable, carboxylic acid functional groups are drawn in the deprotonated state to account for the experimental pH condition.

Herein, we investigated the membrane-disruptive properties of SLL and its molecular building blocks, LA and LacA, using a combination of surface-sensitive measurement approaches in conjunction with various model membrane platforms. Figure 1 presents the molecular structures of the different test compounds, SLL, LA, and LacA along with 12-carbon sodium dodecyl sulfate (SDS)—a widely used industrial surfactant with similar chain properties—that was tested in comparison. Fluorescence spectroscopy measurements were performed to determine the critical micelle concentration (CMC) value of each compound, followed by quartz crystal microbalance–dissipation (QCM-D) and electrochemical impedance spectroscopy (EIS) measurements that enabled real-time tracking of membrane-disruptive interactions. The QCM-D and EIS techniques utilize supported lipid bilayer (SLB) and tethered bilayer lipid membrane (tBLM) platforms, respectively, that possess distinct molecularly engineered features and allow us to study how the various test compounds and mixtures thereof affect membrane mass, viscoelastic, ionic permeability, and capacitance properties during temporally tracked interaction processes. 

In contrast to free-spanning black lipid membranes that form on porous surfaces and lack an underlying solid support, the SLB platform in our experiments consisted of a phospholipid bilayer that is physically adsorbed on top of a hydrophilic silica surface, which provides stability due to attractive noncovalent interactions and enables the use of various surface-sensitive measurement techniques [21]. Likewise, the tBLM platform consisted of a phospholipid bilayer on top of a gold electrode surface and has a low density of longer, tethered lipid molecules (~10 mol%) that form dative bonds with the gold surface to aid physical stability while enabling the formation of a few nm thick ionic reservoir for electrochemical measurements [22]. Compared with other tested fatty acids, monoglycerides, and related surfactants, our findings obtained using these model membrane platforms reveal that SLL, both intact and as a mixture of its hydrolyzed products, exhibits unique patterns of membrane-disruptive behavior in terms of balancing the extent of membrane morphological changes and solubilization that strengthen its application potential. 

## 2. Results and Discussion

### 2.1. Critical Micelle Concentration Determination

**Figure 2:**
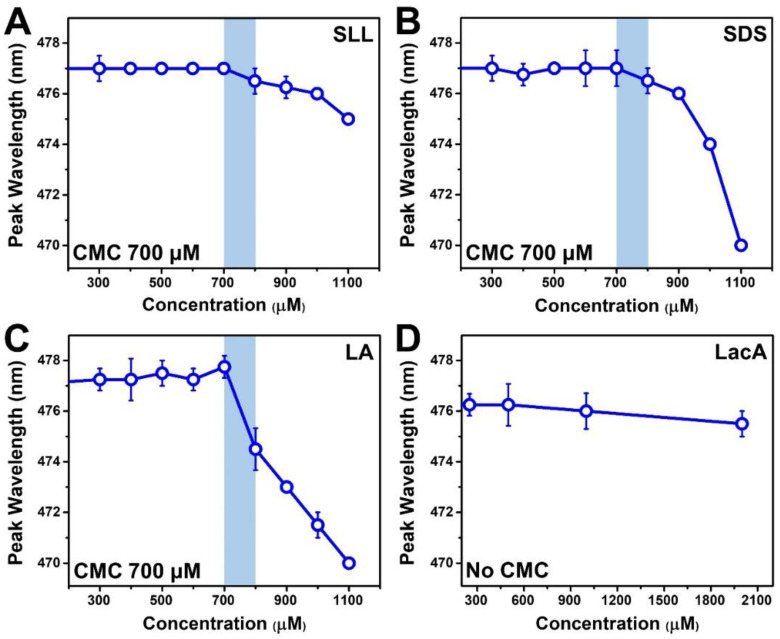
Critical micelle concentration (CMC) of test compounds based on wavelength-shift fluorescence spectroscopy measurements. The highest-intensity (peak) fluorescence emission wavelength of the 1-pyrenecarboxaldehyde (probe molecule) is reported as a function of the test compound concentration in PBS for (**A**) SLL, (**B**) SDS, (**C**) LA, and (**D**) LacA. The mean wavelength values from *n* = 4 measurements are reported, and the error bars represent the corresponding standard deviation values. The shaded region reveals the drop point whereby the CMC is denoted by the highest concentration before the signal drop relative to the data in neat PBS.

The membrane-disruptive characteristics of detergents and surfactants are generally related to molecular surface-active properties [23,24], which led us to evaluate the CMC values of the different test compounds in this study. Indeed, micelles are surface-active macromolecular entities that contribute to the membrane-destabilizing properties of detergents and surfactants [25,26]. Accordingly, the CMC value of a test compound provides insight into the minimum concentration range around which micelles begin to form, hence the degree of potency, i.e., the lowest concentration of a test compound at which membrane destabilization can occur. We performed wavelength-shift fluorescence spectroscopy experiments using 1-pyrenecarboxaldehyde as the probe due to its distinct fluorescence emission properties in aqueous solution and in the hydrophobic interior of micelles [27] (Figure 2). The peak wavelength (maximum-intensity emission) of the probe in PBS was ~477 nm, which agrees with past reports [11,28], and it remained at this value in the presence of monomers only. Upon micelle formation, the probe partitions into the hydrophobic interior of micelles, and the peak emission wavelength decreases accordingly due to the changing dielectric environment surrounding the probe. The compound concentration immediately preceding this wavelength is defined as the CMC because it corresponds to the onset of micelle formation [29].

Following this approach, the CMC values for SLL, SDS, and LA were all determined to be 700 μM (Figure 2A–C). The measured SDS and LA CMC values are consistent with the range of typical values described in past reports [30,31] for similar conditions (e.g., in high salt ionic strength conditions), and all three amphipathic compounds have anionic headgroups and similar chain properties (12-carbon-long, saturated chains), which are important determinants of micellar self-assembly propensity. Note that CMC values can be appreciably higher (by ~6–10-fold) for the same anionic surfactants such as SDS in the case of no/low salt conditions [31,32,33], where intermolecular charge repulsion is greater due to the lack of salt-mediated charge shielding [34], while charge shielding afforded by the physiologically relevant salt concentrations used in this study allowed micellar aggregation to commence at relatively lower concentrations. On the other hand, no CMC was determined for LacA within the tested concentration range, indicating that LacA does not form micelles, and this observation is consistent with its nonamphipathic molecular structure (Figure 2D). Based on the measured CMC values, we defined the concentration range of the test compounds (2000–250 μM in a two-fold dilution series) for QCM-D experiments, which were aimed at directly tracking membrane-disruptive interactions.

### 2.2. QCM-D Tracking of Phospholipid Membrane Interactions

We used the bicelle method [21,35] to fabricate SLB platforms composed of zwitterionic 1,2-dioleoyl-*sn*-glycero-3-phosphocholine (DOPC) lipids on silica-coated QCM-D sensor chips. The resonance frequency (∆f) and energy dissipation (∆D) signals were tracked as a function of time to characterize SLB formation quality and to monitor resulting interactions with added test compounds in monomeric or micellar form (depending on the bulk compound concentration). The ∆f and ∆D signals relate to the hydrodynamically coupled acoustic mass and energy dissipation properties of the adlayer, respectively [36]. SLB formation was confirmed by final ∆f and ∆D shifts of −24.2 ± 0.5 Hz and 0.13 ± 0.09 × 10^−6^, respectively, which were in good agreement with the literature values [37]. Based on the Sauerbrey relationship [38] (∆f ∝ c·∆m, where c is a proportionality constant of −17.7 ng/cm^2^ and ∆m is the mass surface density of adsorbate on the sensor surface; this relationship is valid when ∆D < 1 × 10^−6^), the mean ∆f shift for SLB formation corresponds to a ∆m value of ~428 ng/cm^2^. The small ∆D shift reflects a rigidly attached, nonviscoelastic SLB film, whereas larger ∆D shifts of >1 × 10^−6^ indicate that the adsorbate exhibits more viscoelastic behavior, typically arising from greater hydrodynamically coupled solvent and/or a more flexible architectural configuration of the lipid adlayer [36]. 

Afterwards, the elapsed QCM-D measurement time was reset to zero, and then the test compounds were added at defined bulk concentrations under continuous flow conditions, during which time the QCM-D signals were tracked continuously. The corresponding time-resolved ∆f and ∆D shifts provide insight into changes in the mass and viscoelastic properties of the SLB platform due to compound interactions. For example, a large negative ∆f shift and positive ∆D shift can indicate a membrane morphological change due to shape remodeling (e.g., tubule formation or budding), and various types of membrane morphological changes have been related to the interplay of ∆f and ∆D shifts for a wide range of surfactants/detergents in past studies (additional validation has been performed by fluorescence microscopy [39], localized surface plasmon resonance sensing [40], and molecular dynamics simulations [10]). The measurement responses were tracked throughout the interaction process as well as after a subsequent exchange step with neat buffer until the signals stabilized and/or became negligible (post wash). Upon buffer washing, the compound was removed from the measurement chamber, which allowed us to also assess the final effect of compound treatment on SLB properties. For example, a positive ∆f shift relative to the initial baseline prior to compound addition would indicate lipid mass loss due to membrane disruption of the SLB platform according to the Sauerbrey relationship.

**Figure 3:**
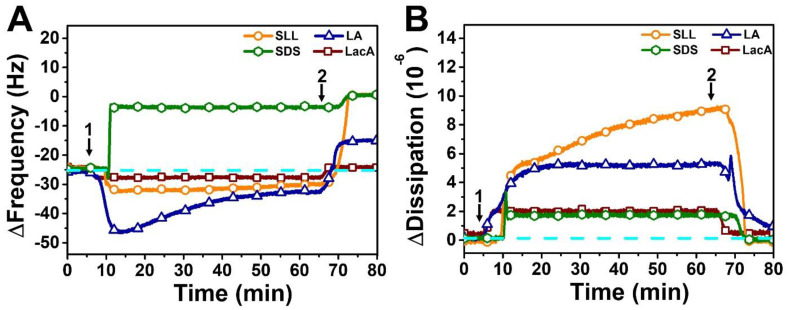
QCM-D tracking of 2000 µM SLL, SDS, LA, and LacA interactions with supported lipid bilayer (SLB) platform. (**A**) Resonance frequency (∆f) and (**B**) energy dissipation (∆D) signals are presented as a function of time. The dashed lines indicate the initial baseline values corresponding to the DOPC SLB platform alone. Arrow 1 specifies the time when test compounds were added (*t* = 5 min) and arrow 2 shows the time when the buffer washing step commenced (*t* = 65 min).

We began by testing the effects of adding 2 mM SDS, SLL, LA, or LacA to the fabricated SLB platform (Figure 3). Upon SDS addition, a rapid increase in the ∆f signal to around −4 Hz (~20 Hz net change) was observed, followed by a buffer washing step that further increased the ∆f signal to around ~0 Hz. The corresponding ∆D signal initially rose to around 2 × 10^−6^ before decreasing to ~0 × 10^−6^ upon buffer washing, and the combination of the ∆f and ∆D shifts indicated that SDS caused complete membrane solubilization (defined as ≥90% decrease in the ∆f shift magnitude post-washing relative to the SLB platform baseline, which is related to the adsorbate mass; conversely, incomplete solubilization is defined as in between an approximately >20% and <90% decrease in the ∆f shift magnitude), as expected [39,41]. Indeed, the final ∆f shift reflects a net change of +24 Hz relative to the SLB platform prior to compound addition, indicating that no adsorbed mass remained on the sensor surface. On the other hand, LA addition caused an initial decrease in the ∆f signal to around −47 Hz before returning to around −33 Hz, and the corresponding ∆D signal stabilized at around 5 × 10^−6^, which is consistent with LA-induced tubule formation [30,39,42]. While the ∆D signal stabilized within 20 min and reflects the viscoelastic properties of the adlayer overall (i.e., due to membrane morphological features and hydrodynamically coupled solvent inside and between the morphological features), the ∆f signal underwent modest changes for a longer time period before stabilizing, which likely reflects slight structural reorganization of the tubule protrusions that form out of the SLB. The final ∆f and ∆D shifts after buffer washing were around −16 Hz (net change of +8 Hz relative to the SLB platform) and ~1 × 10^−6^, respectively, indicating incomplete membrane disruption in the LA case. 

In marked contrast to the aforementioned SDS and LA cases, SLL addition caused only a small decrease in the ∆f signal to around −33 Hz, while there was a large and gradually increasing change in the ∆D signal that reached around 9 × 10^−6^. The longer timescale needed to stabilize the ∆D signal is likely related to structural reorganization of the protrusions formed due to the SLL–membrane interaction, which has also been previously seen with membrane-interacting monoglycerides, in which case small bud protrusions coalesced into larger buds to relieve tension [39]. Interestingly, in the present SLL case, upon buffer washing, the ∆f signal swiftly increased to around ~0 Hz along with a corresponding drop in the ∆D signal to around ~0 × 10^−6^, which indicated that SLL treatment results in complete membrane solubilization. Of note, while SDS caused almost complete membrane solubilization during the initial interaction stage (prior to buffer washing), SLL-induced membrane solubilization occurred only after a subsequent buffer washing step, supporting that SDS is more strongly lytic than SLL. Conversely, LacA caused only small changes in the ∆f and ∆D signals to around −27 Hz and ~2 × 10^−6^ relative to the buffer baseline, respectively, which were fully reversible upon buffer washing and support that LacA by itself only slightly and reversibly interacts with lipid membranes, which is also consistent with its molecular properties, i.e., nonamphipathic character and anionic charge [43]. Collectively, these results demonstrate that SLL has a unique set of membrane-disruptive properties that lie in between those of SDS and LA, which prompted us to further investigate its concentration-dependent interaction behavior.

**Figure 4:**
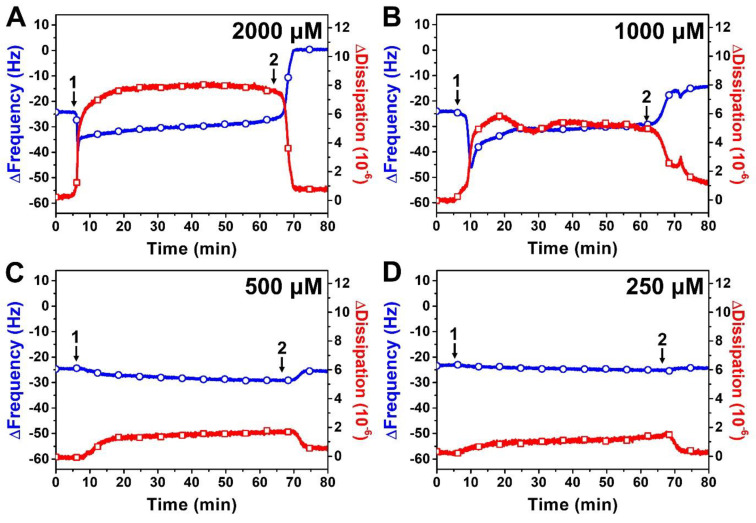
QCM-D tracking of different concentrations of SLL interactions with supported lipid bilayer (SLB) platform. The resonance frequency (∆f) and energy dissipation (∆D) signals are presented as a function of time for (**A**) 2000 μM, (**B**) 1000 μM, (**C**) 500 μM, and (**D**) 250 μM SLL. The initial baseline values correspond to the DOPC SLB platform, while arrow 1 specifies the time when test compounds were added (*t* = 5 min) and arrow 2 shows the time when the buffer washing step commenced (*t* = 65 min).

Figure 4 presents QCM-D measurement responses when different SLL concentrations were added to DOPC SLB platforms. As mentioned above, the addition of 2000 μM SLL caused an initial decrease in the ∆f shift from −24 Hz to around −39 Hz, and the signal gradually stabilized at around −33 Hz (Figure 4A). The corresponding ∆D shift increased from ~0 × 10^−6^ and stabilized around 8 × 10^−6^, while buffer washing caused the ∆f and ∆D signals to change to around ~0 Hz (net change of +24 Hz relative to SLB platform baseline) and ~1 × 10^−6^, respectively, indicating complete solubilization. Similar interaction behavior occurred for 1000 μM SLL, with an initial drop in the ∆f signal to around −45 Hz that then gradually climbed and stabilized at around −33 Hz (Figure 4B). The drop was accompanied by an increase in the ∆D signal to around ~6 × 10^−6^, and subsequent buffer washing caused the ∆f and ∆D signals to shift to around ~11 Hz (net change of +13 Hz relative to SLB platform baseline) and ~1.8 × 10^−6^, respectively. Hence, 1000 μM SLL treatment still caused extensive membrane disruption at this concentration but not complete membrane solubilization as in the 2000 μM SLL case.

While ≥1000 μM SLL concentrations are above the SLL CMC in PBS (~700 μM) and hence SLL is present in micellar form in those cases, we also proceeded to test lower SLL concentrations below the CMC, i.e., when SLL is present in monomeric form only. The addition of 500 μM SLL monomers caused only a minor decrease in the ∆f signal, down to around −27 Hz (~3 Hz decrease), along with a modest increase in the ∆D signal to around ~1.5 × 10^−6^ (Figure 4C). The ∆f and ∆D signals returned to around −24 Hz and 0.6 × 10^−6^, respectively, after buffer washing. These shifts indicate that SLL monomers had only slight and reversible interactions with the lipid membrane. The same pattern of membrane-interaction behavior was observed upon 250 μM SLL addition, in which case there were only minor and reversible changes in the ∆f and ∆D signals to around −26 Hz (~2 Hz decrease) and ~1.3 × 10^−6^, respectively (Figure 4D). Altogether, the QCM-D data support that SLL disrupts lipid membranes in a CMC-dependent manner [44], whereby ≥1000 μM SLL concentrations caused extensive membrane disruption, including complete solubilization at sufficiently high concentrations, while lower SLL concentrations had negligible impact on lipid membrane properties. 

**Figure 5:**
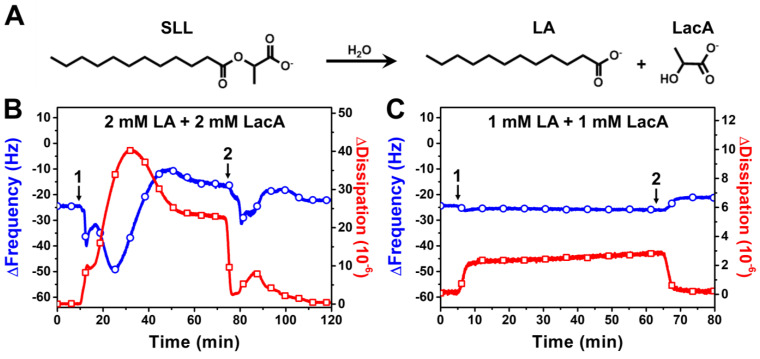
QCM-D tracking of SLL hydrolytic cleavage product interactions with supported lipid bilayer (SLB) platform. (**A**) Molecular structures of SLL and its hydrolytic products, LA and LacA. The resonance frequency (∆f) and energy dissipation (∆D) signals are presented as a function of time for (**B**) 2000 μM LA + 2000 μM LacA and (**C**) 1000 μM LA + 1000 μM LacA. The initial baseline values correspond to the DOPC SLB platform, while arrow 1 specifies the time when test compounds were added (*t* = 5 min) and arrow 2 shows the time when the buffer washing step commenced (*t* = 65 min).

SLL has been reported to exhibit antimicrobial activity in biological environments [15,19], in which the ester bond in SLL is potentially susceptible to hydrolytic cleavage. Therefore, we also performed QCM-D experiments to investigate the membrane-disruptive effects of SLL hydrolytic breakdown products, i.e., a mixture of free LA and LacA, on the DOPC SLB platform (Figure 5). The hydrolytic cleavage of the ester bond in SLL can occur upon reaction with a water molecule, which can be catalyzed by enzymes in biological environments, for example, as described in [16], and yields one LA and one LacA molecule (Figure 5A). Therefore, we tested the membrane-disruptive effects of 2000 μM LA + 2000 μM LacA (equivalent to 2000 μM SLL) and 1000 μM LA + 1000 μM LacA (equivalent to 1000 μM SLL).

The addition of 2000 μM LA + 2000 μM LacA resulted in a swift and transient drop in the ∆f signal to around −50 Hz before transitioning upwards to around −8 Hz with complex, nonmonotonic behavior during the interaction stage (Figure 5B). The interaction also caused the ∆D signal to rise up to around 41 × 10^−6^, which then decreased and stabilized at around 23 × 10^−6^ during this stage. The maximum ∆D shift caused by the mixture was notably larger than that caused by SLL itself and indicates that the transient interaction involved extensive membrane morphological changes to respond to compound-induced membrane strain. Strikingly, however, the final ∆f and ∆D shifts, after a subsequent buffer washing step until reaching stabilization, were around −23 Hz and 0.4 × 10^−6^, respectively, which are similar to the baseline SLB values and demonstrate that the LA + LacA mixture effects were mainly reversible and caused only minor changes in the final lipid membrane properties, i.e., the lipid bilayer remained largely intact as judged by the final QCM-D responses post-washing. Moreover, the addition of 1000 μM LA + 1000 μM LacA caused only a modest drop in the ∆f and ∆D signals to around −27 Hz and 2.8 × 10^−6^, respectively, which returned to around −21 Hz and 0.2 × 10^−6^, respectively, after a buffer washing step. The latter result indicates that 1000 μM LA + 1000 μM LacA behaves similarly to 1000 μM LA alone [39], while the case of 2000 μM LA + 2000 μM LacA is particularly interesting, because the data support that this mixture causes more extensive membrane morphological changes/remodeling than 2000 μM LA alone. Hence, it is likely that the LacA membrane interaction, even if minor, plays some type of potentiating role to facilitate membrane remodeling during the interaction process. 

**Figure 6:**
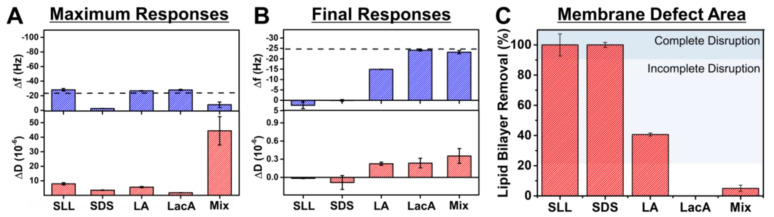
Summary of QCM-D measurement responses related to phospholipid membrane interactions. (**A**) Maximum changes in resonance frequency (∆f) and energy dissipation (∆D) signals during interaction process and corresponding (**B**) final ∆f and ∆D shifts after subsequent buffer washing step due to 2000 μM compound/mixture interactions with supported lipid bilayer (SLB) platform. Dashed line corresponds to typical SLB values and Mix refers to 2000 µM LA + 2000 µM LacA. (**C**) Membrane solubilization extent due to compound interaction based on data in panel (**B**). The mean values are reported from at least *n* = 3 measurements, and the error bars represent the corresponding standard deviation values. Shaded regions indicate extents of lipid bilayer removal that correspond to complete or incomplete membrane disruption based on the defined quantitative criteria.

Based on the QCM-D data, we further analyzed trends in the maximum signal responses during the interaction process and in the final responses after buffer washing for 2000 µM concentrations of each compound and of the mixture (Figure 6). Note that the mixture concentration was 2000 µM LA + 2000 µM LacA, which is equivalent to 2000 µM SLL. We focused on the maximum responses because they correspond to the extent of membrane morphological changes during the interaction process (i.e., largest absolute ∆f and ∆D shifts during the compound addition stage relative to the SLB baseline values) and on the final responses (i.e., final ∆f and ∆D shifts relative to the SLB baseline values) because they correspond to the resulting extent of membrane disruption. The latter values were also converted into quantitative estimates of membrane solubilization based on the Sauerbrey relationship, as described above. In terms of maximum responses, SLL, LA, and LacA had only minor effects on the ∆f signal, while SLL and LA caused modest ∆D shifts unlike in the LacA case, which was only minor (Figure 6A). By contrast, SDS directly caused membrane solubilization and, in marked contrast to all of these individual compound cases, the LA + LacA mixture caused a modest ∆f shift increase and a large ∆D shift increase, which indicates extensive membrane morphological changes, i.e., shape remodeling, during the interaction stage. These analyses were further verified by plotting time-independent curves of the ∆f and ∆D signals, which indicated that the LA + LacA mixture caused the most extensive changes in adlayer properties, followed by SLL and LA, whereas SDS caused direct solubilization and LacA had nearly negligible effect (Figure S1).

The final responses also indicate that SLL and SDS both caused complete membrane solubilization, while LacA and the LA + LacA mixture had only minor effects on membrane properties. LA had intermediate effects and caused modest membrane disruption but only incomplete membrane solubilization (Figure 6B). The extent of membrane solubilization was further estimated based on the amount of lipid bilayer mass removed from the sensor surface (since ∆m is related to the final ∆f shift relative to the SLB platform baseline value according to the Sauerbrey equation, as described above; see also, e.g., representative data of overtone-independent responses indicating rigid adlayer properties in Figure S2). It was concluded that SLL and SDS caused ~100% solubilization. On the other hand, LA and the LA + LacA mixture caused around 41 ± 1% and 5 ± 2% solubilization, respectively, while LacA caused negligible solubilization (~0%) (Figure 6C).

**Figure 7:**
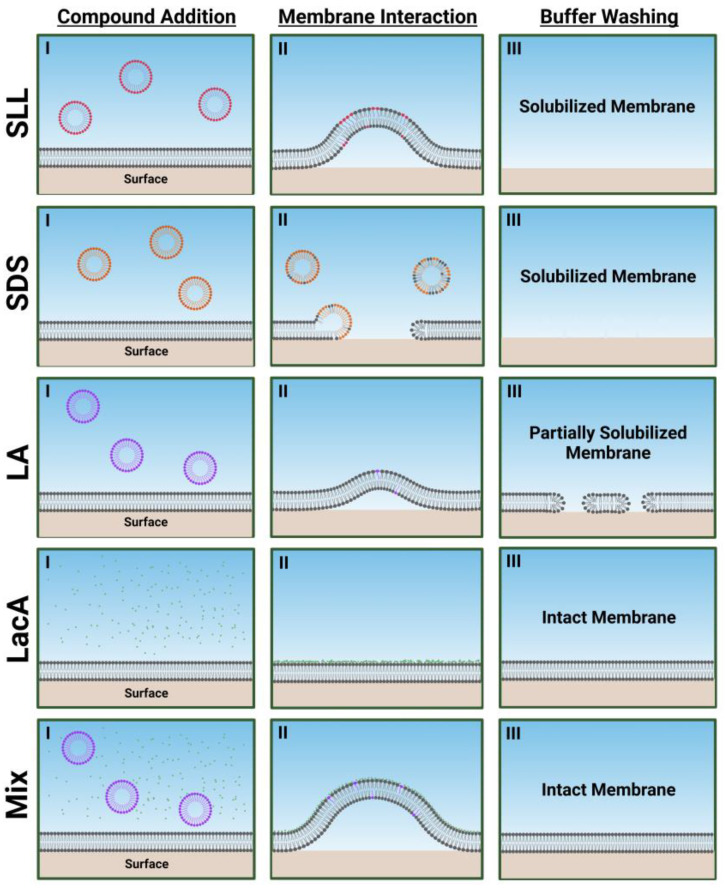
Schematic summary of membrane-disruptive interactions of compounds and mixtures with supported lipid bilayer (SLB) platform. Proposed mechanisms are based on QCM-D data interpretation in terms of maximum responses during interaction processes and final responses after buffer washing for 2000 µM concentrations of each compound or mixture. I, II, III correspond to initial compound addition to the SLB platform, membrane morphology during the transient membrane interaction process, and final membrane morphology after buffer washing step, respectively. The schematic illustrations in (II) are artistic renderings based on the relative extent of membrane morphological changes/shape remodeling that are reflected in the QCM-D measurement responses, whereby larger maximum responses correspond to greater morphological changes trendwise (Mix > SLL > LA). Note that the mixture concentration is 2000 µM LA + 2000 µM LacA, which is equivalent to 2000 µM SLL.

Figure 7 presents schematic illustrations describing how each compound and mixture disrupts the SLB platforms based on the trends in QCM-D data. SLL caused extensive membrane morphological changes (possibly related to coalescence of smaller morphological protrusions into larger ones over time as discussed above) and ultimately resulted in complete membrane solubilization upon buffer washing. This behavior fits in between the direct and rapid membrane solubilization caused by SDS and the more modest membrane morphological changes and partial membrane solubilization caused by LA. By contrast, LacA alone had negligible membrane-disruptive effects, and the LA + LacA mixture caused extensive membrane morphological changes during the interaction process itself, but membrane solubilization did not occur, and the SLB remained largely intact. The effects of the LA + LacA mixture, i.e., large, transient morphological changes but limited solubilization compared with LA alone, are striking, because they are nearly opposite to previously observed trends with LA + glycerol monolaurate (GML) mixtures that instead caused synergistic membrane disruption and irreversible solubilization compared with LA or GML alone [30]. This difference highlights the potential of designing different mixtures with distinct functionalities; for example, the LA and GML mixture has two membrane-disrupting, antimicrobial lipids with amphipathic properties, while the LA and LacA mixture has one membrane-disrupting, antimicrobial lipid with amphipathic properties and one nonamphipathic molecule.

### 2.3. EIS Characterization of Membrane-Disruptive Interactions

Complementing the QCM-D experiments, we also performed EIS experiments to track changes in the conductance (G_m_) and capacitance (C_m_) signals of DOPC tBLM platforms in response to compound or mixture addition as a function of time. G_m_ is directly proportional to the tendency of ions to flow through the tethered lipid bilayer, whereas C_m_ is inversely related to membrane thickness [45,46,47]. The tBLM fabricated on the gold electrode was characterized using EIS to confirm its formation according to reference values [48,49], and the measured signals served as a baseline before 2000 µM compounds/mixtures were added to the tBLM platform for 30 min, which preceded a subsequent buffer washing step. Note that the baselines of G_m_ and C_m_ values for tBLM formation were in the acceptable range of <~3 μS and 0.7–1.2 μF/cm^2^, respectively. 

**Figure 8:**
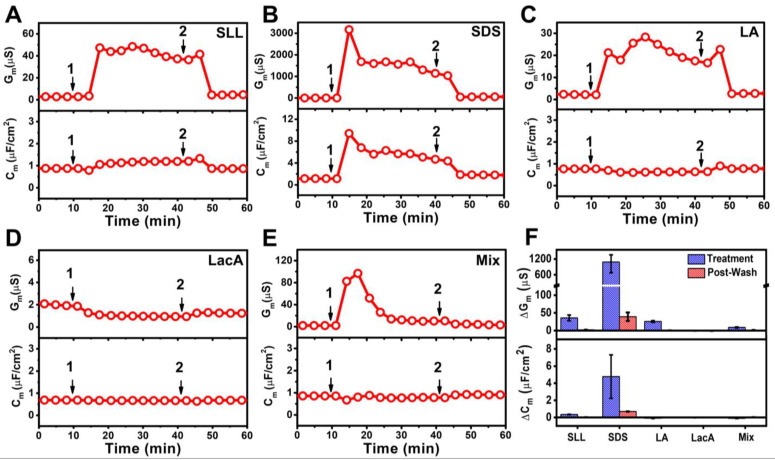
EIS monitoring of 2000 µM (**A**) SLL, (**B**) SDS, (**C**) LA, (**D**) LacA, and (**E**) LA + LacA (Mix) interactions with tethered bilayer lipid membrane (tBLM) platform. Time-resolved changes in the conductance (G_m_) and capacitance (C_m_) signals were tracked. The initial baseline values correspond to the tBLM platform prior to compound/mixture addition. The test compounds/mixtures were added at *t* = 10 min (arrow 1) and a buffer washing step was performed at *t* = 40 min (arrow 2). Note that the mixture concentration is 2000 µM LA + 2000 µM LacA, which is equivalent to 2000 µM SLL. (**F**) Summary of G_m_ and C_m_ shifts after compound/mixture addition for 30 min (Treatment) and after buffer washing (Post-Wash). Data are reported as mean values from *n* = 3 measurements, and the error bars represent the corresponding standard deviation values.

Figure 8 displays the time-resolved G_m_ and C_m_ signals for all experimental series. The addition of 2000 µM SLL resulted in a modest increase in and rapid stabilization of the G_m_ and C_m_ signals around 47 μS and 1.2 μF/cm^2^, respectively (Figure 8A). After buffer washing, the G_m_ and C_m_ signals decreased to around 4.7 μS and 0.9 μF/cm^2^, respectively. In marked contrast, the addition of 2000 µM SDS caused appreciably greater membrane disruption, as indicated by peak G_m_ and C_m_ values of around 3200 μS and 9.4 μF/cm^2^, respectively, which gradually stabilized at around 1400 μS and 5.7 μF/cm^2^, respectively (Figure 8B). Subsequent buffer washing caused a decrease in the measurement signals to a G_m_ value of around 58 μS and a C_m_ value of around 1.8 μF/cm^2^. Notably, the final G_m_ shift value was still >10-fold greater than in the SLL case, indicating a greater increase in membrane permeability.

On the other hand, when 2000 µM LA was added to the tBLM platform, the G_m_ and C_m_ signals increased more modestly to and stabilized at around 25 μS and 0.6 μF/cm^2^, respectively. Afterwards, the buffer washing step caused a decrease in the G_m_ and C_m_ signals to near-baseline values of 2.7 μS and 0.8 μF/cm^2^, respectively (Figure 8C). The effect of adding 2000 µM LacA was even more modest, leading to only a slight decrease in the G_m_ signal by around 1.0 μS and a nearly negligible decrease in the C_m_ signal by around 0.7 μF/cm^2^ (Figure 8D). The G_m_ and C_m_ signals remained nearly unchanged after buffer washing and reached values of around 1.2 μS and 0.7 μF/cm^2^, respectively. The addition of the 2000 µM LA + 2000 µM LacA mixture had a more pronounced effect on membrane permeability and caused a transient increase in the G_m_ signal to around 97 μS before gradually decreasing to around 12 μS (Figure 8E). The corresponding C_m_ signal decreased slightly to around 0.8 μF/cm^2^. The G_m_ and C_m_ values were around 3.1 μS and 0.9 μF/cm^2^ after buffer washing, respectively, which indicated modest membrane disruption treatment effects that were comparable to those of SLL.

Figure 8F summarizes the net G_m_ and C_m_ shifts relative to measurement baselines, which correspond to the responses during the interaction process after introducing the compounds/mixture for a 30 min elapsed time period (Treatment) and after buffer washing (Post-Wash) due to molecular-level interactions with the tBLM platform. SLL addition caused G_m_ and C_m_ shifts of 36 ± 8 μS and 0.3 ± ~0 μF/cm^2^, respectively, and the shifts decreased to 2.2 ± 0.2 μS and 0 μF/cm^2^, respectively, after buffer washing. By contrast, SDS addition caused G_m_ and C_m_ shifts of around 1051 ± 395 μS and 4.8 ± 2.5 μF/cm^2^, respectively. In that case, the final G_m_ and C_m_ shifts decreased to around 39 ± 12 μS and ~0.7 μF/cm^2^, respectively, after buffer washing. LA addition caused the G_m_ shift to increase to 26 ± 2 μS upon treatment and then decrease back down to 0.4 ± 0.1 μS after buffer washing, whereas the C_m_ shifts were ~0 μF/cm^2^ in both cases. Distinct from the other cases, LacA addition caused a slightly negative G_m_ shift decrease of around −1.2 ± 0.1 μS upon treatment that slightly increased to −1.1 ± 0.1 μS after washing, while there were again negligible C_m_ shifts of ~0 μF/cm^2^. Interestingly, treatment with the LA + LacA mixture caused a rapid spike in the G_m_ signal up to around ~100 μS relative to the baseline that dropped to and stabilized at around 8.8 ± 1.6 μS during the interaction process. The G_m_ shift further decreased to around 1.4 ± 0.2 μS after buffer washing, and the corresponding C_m_ shifts were negligible at around ~0 μF/cm^2^. 

Together, these findings support that SDS caused the largest G_m_ and C_m_ shifts, while the relatively steady G_m_ shifts after compound addition indicate that SLL, SDS, and LA mainly caused transient membrane defect formation. Interestingly, the LA + LacA mixture induced a more dynamic response as there was a rapidly occurring membrane-disruptive effect that dissipated over time. Pronounced membrane-disruptive effects remained after buffer washing only in the SDS and SLL cases (and to a much greater extent in the SDS case; the SLL effect was still relatively minor), while LA, LacA, and the LA + LacA mixture had reversible or negligible effects. 

**Figure 9:**
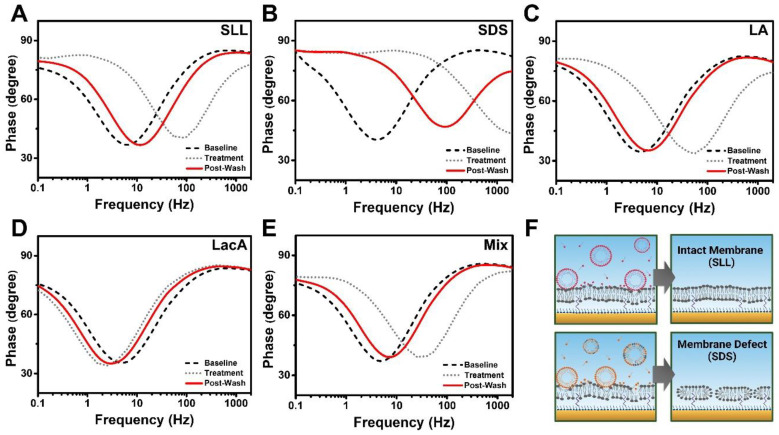
Bode plot analysis of EIS measurement data. The plots and phase shifts of the tBLM platform due to interactions are presented for (**A**) SLL, (**B**) SDS, (**C**) LA, (**D**) LacA, and (**E**) LA + LacA (Mix). Changes in membrane conditions upon compound addition (Treatment) and after buffer washing (Post-Wash) were evaluated from plot shifts compared with the original state of membranes (Baseline). (**F**) Schematic illustrations of interactions between the tBLM platform and SLL or SDS upon compound addition and in the final state after buffer washing based on the Bode plots. SLL modestly disrupted the tBLM platform in a more transient manner, but the membrane remained intact (**top panel**), while SDS removed lipids from the membrane and caused permanent membrane defect formation (**bottom panel**).

In the EIS measurements, we also analyzed the phase angle over the set frequency range, which allows the data to be represented in time-dependent Bode plots of the phase angle vs. the logarithm of the frequency. The Bode plot format is widely used to interpret tBLM measurement data obtained from the experimental setup used in this study, so it was selected for aiding data interpretation and comparison with the literature references, while other visualizations of EIS data such as the Nyquist plot are also possible [50]. In the Bode plot format, the phase value at the phase minima shows the ratio of capacitances between the gold electrode and the tBLM, while the frequency at the phase minima indicates the conductance [51,52]. As such, changes in electrochemical properties of the tBLM platform can be ascertained and compared from phase minima shifts using Bode plots (Figure 9). For SLL, the phase minima shifted to larger frequencies during treatment, while the minima returned to lower frequencies after buffer washing (Figure 9A). By contrast, SDS induced a phase minimum shift to a higher phase angle and much larger frequencies due to appreciable increases in both membrane conductance and capacitance (Figure 9B). After buffer washing, the phase minima only partially shifted back to lower frequencies and did not return to the baseline value, which is indicative of the presence of irreversible membrane defects.

In addition, LA treatment exhibited similar membrane-disruptive effects to those of SLL treatment, while exhibiting near-baseline recovery of the measurement signature after buffer washing (Figure 9C). In the LacA case, the phase minima shifted to slightly lower frequencies than the baseline, suggesting minor changes in the tBLM properties akin to membrane thinning (Figure 9D). On the other hand, the LA + LacA mixture transiently disrupted the tBLM platform to a greater extent, which bears resemblance to the LA case (Figure 9E). This similarity is particularly noteworthy because the magnitude of the transient G_m_ signal increase was greater in the mixture case, but the Bode plots indicate that both LA and LA + LacA induce mainly reversible membrane damage. Based on the time-resolved EIS data and Bode plot analysis, Figure 9F depicts the proposed molecular-level interactions of SLL and SDS with the tBLM platforms and corresponding effects on membrane integrity. At 2 mM SLL concentration, which is above its CMC, SLL molecules interact with the top phospholipid layer of the tBLM and disrupt membrane packing by inserting into the membrane but do not appear to be able to form solubilizing phospholipid–micelle complexes, i.e., extensive solubilization does not occur. As such, the characterization data support that weakly interacting SLL molecules were removed by buffer washing, and the disrupted phospholipid molecules reassembled to form a largely intact membrane. By contrast, SDS has a relatively smaller headgroup, and its micelles can form solubilizing phospholipid–detergent complexes that cause phospholipid removal from the membrane [47,53]. Consequently, the partial phospholipid removal caused by SDS elicited an increase in tBLM permeability and resulted in irreversible membrane defect formation.

Together, the EIS results indicate that SDS had the strongest membrane-disruptive effects, while SLL exhibited an intermediate degree of membrane disruption that lays between those of SDS and LA, as indicated by the time-resolved interaction kinetics. On the other hand, LacA had only minor effects on tBLM properties but did cause slight membrane thinning that appeared to enhance the membrane-disruptive interactions between LA in the LA + LacA mixture and the tBLM platform. Interestingly, in terms of final treatment outcomes post-washing, SLL and LA did not cause permanent membrane damage to the tBLM platform, which is likely related to its more flexible nanoarchitecture compared with the more rigidly attached SLB platform in the QCM-D experiments, in which case both compounds caused extensive, irreversible membrane disruption. Indeed, the tBLM platform is only sparsely tethered to the underlying substrate, which enables greater structural flexibility to respond to strain-related membrane interactions, as compared with SLB platforms that conformally coat substrates. 

From a broader perspective, these findings agree well with the overall trend in the QCM-D data showing that SDS caused the greatest membrane damage, followed by SLL, LA, and LacA in decreasing order. Moreover, the QCM-D data also showed that the LA + LacA mixture caused extensive, albeit reversible, membrane morphological changes as the compounds interacted with the SLB but did not trigger membrane solubilization, even after a buffer washing step. As such, while the QCM-D and EIS techniques rely on different sensing principles and utilized distinct types of model membrane platforms, the resulting data complement one another and provide mechanistic insight into the membrane-disruptive properties of SLL and its hydrolytic product mixture, i.e., in the LA + LacA mixture, LacA impacted membrane properties in a manner that enhanced LA-induced membrane morphological changes but did not affect the resulting extent of membrane disruption. 

## 3. Conclusions and Outlook

In recent years, there have been growing efforts to elucidate how antimicrobial lipids such as medium-chain fatty acids and monoglycerides disrupt phospholipid membranes from a biophysical perspective. This membrane biophysics approach complements classical biological assays that typically measure the degree of pathogen inhibition by providing insight into corresponding membrane interaction processes, which has proven critical to understanding how fatty acids and monoglycerides exhibit distinct mechanisms of membrane disruption. Importantly, the biophysical viewpoint has helped to shift from empirically testing individual antimicrobial lipid compounds to drawing out structure–function relationships in terms of how compound properties such as chain length and headgroup charge affect potency and mechanism of action. More recently, it has been possible to apply biophysical measurement strategies to develop synergistic mixtures of antimicrobial lipids and to test industrial detergents with antimicrobial properties. 

Within the latter scope, lactylates such as SLL are promising antimicrobials that have demonstrated utility in food science and agricultural applications so far, but there is limited biophysical understanding about how they disrupt phospholipid membranes. Our QCM-D and EIS measurements address this need while building a broader picture of how SLL functions relative to other widely studied antimicrobial lipids, surfactants, and detergents such as LA and SDS. To this end, a key objective in the field is identifying surfactant-like molecules that potently disrupt phospholipid membranes but are not fully indiscriminate. For example, SDS generally causes a high degree of membrane solubilization, but this indiscriminate effect may be disadvantageous for in vivo translational applications. On the other hand, LA causes partial membrane disruption to a much smaller extent, which may be less desirable as well. 

Interestingly, SLL fits in between the SDS and LA cases and demonstrates a high degree of membrane solubilization in some cases but is sensitive to the specific membrane nanoarchitecture (e.g., complete solubilization of the SLB platform but reversible interactions with the tBLM platform was observed, potentially due to differences in the membrane flexibility and corresponding effects on responding to interaction-related membrane strain), which implies that it might be possible to utilize SLL or rationally engineered derivatives to disrupt small, membrane-enveloped virus particles but not much larger, human cell membranes, for example. By utilizing biophysical measurement platforms, it becomes possible to explore such options and to more broadly harness the potential of tuning antimicrobial lipid and related detergent/surfactant interactions with phospholipid membranes in order to modulate the potency and mechanistic details of membrane disruption, especially in combination with tailoring biodegradation profiles and utilizing nanoscale delivery strategies [54].

## 4. Materials and Methods 

### 4.1. Reagents

Sodium lauroyl lactylate (SLL) was purchased from Henan Tainfu Chemical Co., Ltd. (Zhengzhou, China). Sodium dodecyl sulfate (SDS), lauric acid (LA), lactic acid (LacA), and 1-pyrenecarboxaldehyde were obtained from Sigma-Aldrich (St. Louis, MO, USA). 1,2-Dihexanoyl-*sn*-glycero-3-phosphocholine (DHPC) and 1,2-dioleoyl-*sn*-glycero-3-phosphocholine (DOPC) were purchased from Avanti Polar Lipids, Inc. (Alabaster, AL, USA). Phosphate-buffered saline (PBS, pH 7.4) was obtained from Gibco (Carlsbad, CA, USA). All aqueous samples were prepared using Milli-Q-treated water (MilliporeSigma, Burlington, MA, USA) with a minimal electrical resistivity of >18 MΩ·cm.

### 4.2. Sample Preparation

SLL, LA, and LacA were initially dissolved in ethanol at 200 mM compound concentration, before each compound was diluted 100-fold with PBS in order to prepare 2 mM stock solutions, while the 2 mM SDS stock was made by directly adding the measured amount of SDS to the same solvent system. Immediately before experiments, the stock solutions were vortexed moderately and then incubated at 70 °C for 30 min. Subsequent dilutions in PBS were made in two-fold incremental steps to prepare test samples.

### 4.3. Critical Micelle Concentration (CMC) Assay

The CMC values of the test compounds were determined by wavelength-shift fluorescence spectroscopy experiments using a SpectraMax iD5 microplate reader (Molecular Devices, San Jose, CA, USA), as previously described [31]. Various concentrations of each compound were mixed with 0.1 µM 1-pyrenecarboxaldehyde (365.5 nm excitation wavelength), which served as the fluorescent probe that can partition into the hydrophobic interior of micelles when present, and its maximum-intensity fluorescence emission wavelength varies depending on the local dielectric environment. Accordingly, the fluorescence emission spectrum was measured in the range of 410 nm to 600 nm for each sample. To determine the corresponding CMC value of each compound, a plot of the maximum-intensity fluorescence emission wavelength was constructed as a function of compound concentration. At least four technical replicates were measured per sample.

### 4.4. Quartz Crystal Microbalance–Dissipation (QCM-D)

To investigate the membrane-disruptive interactions of the test compounds against a supported lipid bilayer (SLB) coating, a Q-Sense E4 instrument (Biolin Scientific AB, Gothenburg, Sweden) was used for the QCM-D measurements, as previously described [39]. During measurements, time-resolved changes in the resonance frequency (∆f) and energy dissipation (∆D) signals of the SLB-coated sensor chips were measured in order to track the mass and viscoelastic properties of the SLB film, respectively. For all experiments, silica-coated sensor chips (model no. QSX 303, Biolin Scientific AB) were used, because SLBs can readily form in situ on silica surfaces. Prior to experiment, the silica-coated sensor chips were cleaned by using deionized water and ethanol rinses, followed by nitrogen gas drying and oxygen plasma treatment with a CUTE-1MPR machine (Femto Science Inc., Hwaseong, Republic of Korea). Once the sensor chips were assembled in the measurement flow cells, all liquid exchange steps were controlled by a peristaltic pump (Reglo Digital, Ismatec, Glattbrugg, Switzerland), and the volumetric flow rate was fixed at 50 μL/min. Data collection and analysis were managed by the QSoft401 (Biolin Scientific AB; version no. 2.5.28.732) and QTools (Biolin Scientific AB; version no. 3.1.33.567) software packages. Measurement data were collected at multiple odd overtones, and reported data are from the fifth overtone unless otherwise noted.

### 4.5. Electrochemical Impedance Spectroscopy (EIS)

EIS measurements tracking the effects of the test compounds on the electrochemical properties of tethered bilayer lipid membrane (tBLM) platforms were conducted in alternating current (AC) mode by using a tethaPod instrument (product code: SDx-R1, SDx Tethered Membranes, Sydney, Australia), as previously described [47]. The AC excitation value was 25 mV, no offset voltage was applied, and the swept frequency range was 0.1 Hz to 2000 Hz. During measurements, time-resolved changes in the conductance (G_m_) and capacitance (C_m_) signals of the tBLM-coated gold electrode chips were measured in order to track the ionic charge passing through and electrical charge buildup in the tBLM platform, respectively. To fabricate tBLMs, a precoated gold electrode surface (product code: SDx-BG, SDx Tethered Membranes) that had 90% spacer (hydroxyl terminated benzyldisulphide tetra-ethylene glycol) and 10% tether (benzyldisulphide polyethylene glycol phytanyl) molecules on the sensor surface was first assembled in a six-chamber tethaPlate cartridge (product code: SDx-T10, SDx Tethered Membranes). Then, a DOPC lipid coating was formed on top of the tethered monolayer by the solvent-exchange method in order to complete tBLM formation, as previously described [47]. To assess tBLM platform quality, G_m_ and C_m_ values of <~3 μS and 0.7–1.2 μF/cm^2^, respectively, were considered to be suitable baseline values. Data collection was managed by the tethaQUICK software package (product code: SDx-B1, SDx Tethered Membranes; version no. 2.0.58).

## Data Availability

The data presented in this study are available upon reasonable request from the corresponding authors.

## Supplementary Materials

The following supporting information can be downloaded at: https://www.mdpi.com/article/10.3390/ijms24119283/s1.
