# Supplementary material for: Unraveling Membrane-Disruptive Properties of Sodium Lauroyl Lactylate and Its Hydrolytic Products: A QCM-D and EIS Study
Source: Int J Mol Sci. 2023 May 25;24(11):9283. doi: 10.3390/ijms24119283 (PMC10253177; doi:10.3390/ijms24119283)
Supplement: Supplementary file 1 [file ijms-24-09283-s001.zip › ijms-2370201-supplementary.pdf]

**Supplementary Information**

# Unraveling Membrane-Disruptive Properties of Sodium Lauroyl Lactylate and Its Hydrolytic Products: A QCM-D and EIS Study

**Negin Gooran [1], Sue Woon Tan [1], Bo Kyeong Yoon [2,*] and Joshua A. Jackman [1,*]**

[1] School of Chemical Engineering and Translational Nanobioscience Research Center, Sungkyunkwan University, Suwon 16419, Republic of Korea

[2] School of Healthcare and Biomedical Engineering, Chonnam National University, Yeosu 59626, Republic of Korea

* Correspondence: bkyoon@jnu.ac.kr (B.K.Y.); jjackman@skku.edu (J.A.J.)

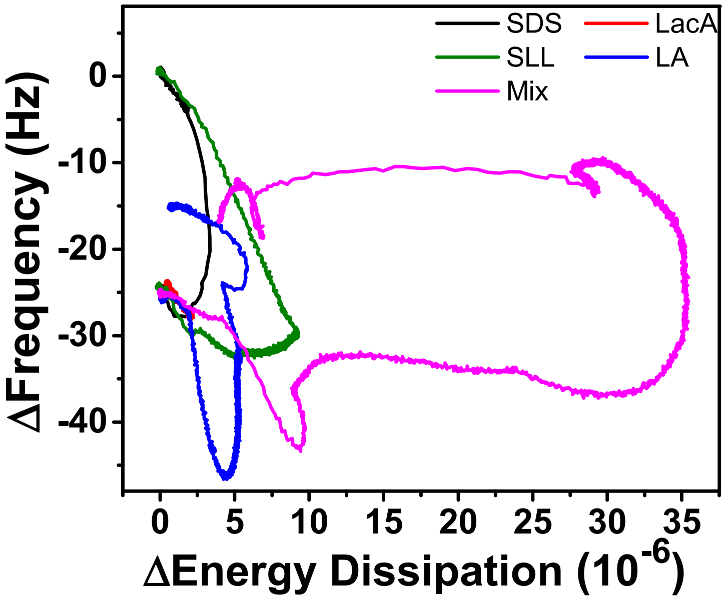


**Figure S1.** Time-independent analysis of QCM-D resonance frequency (Δf) and energy dissipation (ΔD) signals for membrane-disruptive interactions of 2000 µM SLL, SDS, LA, LacA and Mix (2000 µM LA + 2000 µM LacA) with supported lipid bilayer (SLB) platform. Data correspond to results described in Figures 3 and 5B.

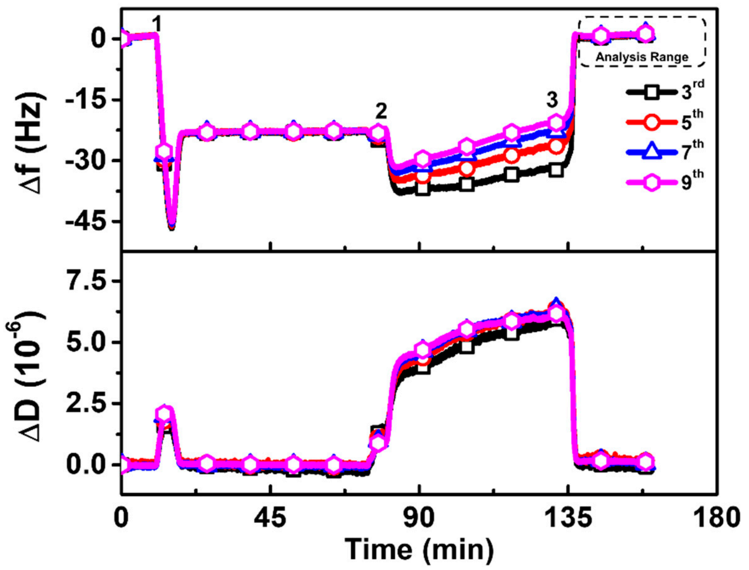


**Figure S2.** QCM-D tracking of 2000 μM SLL interaction with supported lipid bilayer (SLB) platform and corresponding overtone analysis. The resonance frequency (Δf) and energy dissipation (ΔD) signals at different odd overtones are presented as a function of time. The initial baseline values correspond to the silica-coated QCM-D sensor surface in buffer while Arrows 1, 2, and 3 correspond to bicelle addition for SLB formation, SLL addition to the SLB platform, and a buffer washing step. The Sauerbrey model was applied to analyze the amount of lipid mass remaining on the sensor surface after the buffer washing step (relative to the amount corresponding to the fabricated SLB platform; see boxed-in region) when the measurement responses displayed overtone-independent behavior and had low energy dissipation.
